# Gut microbiota and immunometabolism in obesity

**DOI:** 10.1080/19490976.2026.2667610

**Published:** 2026-05-05

**Authors:** Alba Torres-Mayo, Rebeca Liébana-García, Marta Olivares, Aize Pellón, Juan Anguita, Yolanda Sanz

**Affiliations:** a Microbiome Innovation in Nutrition and Health Research Unit (INNOBIOME), Institute of Agrochemistry and Food Technology, Spanish National Research Council (IATA-CSIC), Valencia, Spain; b Food Science, Technology and Management PhD Program, Universitat Politècnica de València, Valencia, Spain; c Inflammation and Macrophage Plasticity Laboratory, CIC bioGUNE-BRTA (Basque Research and Technology Alliance), Derio, Spain; d Ikerbasque, Basque Foundation for Science, Bilbao, Spain

**Keywords:** Gut microbiota, immunometabolism, diet, obesity

## Abstract

The gut microbiota plays a central role in modulating both immunity and metabolism. Obesity-associated microbiota configuration is a critical driver of persistent inflammatory activation and immune dysfunction, ultimately leading to chronic metabolic disorders. Immunometabolism examines how metabolic demands shape immune cell function and how immune responses influence cellular metabolism. Emerging research on how the gut microbiota contribute to immune cell metabolic processes and the resulting health outcomes is deepening our understanding of the mechanisms underlying obesity and metabolic diseases. In this review, we summarize how intracellular metabolic pathways and master regulators, such as mTOR and AMPK, orchestrate immune cell function and how their dysregulation contributes to obesity-associated immune and metabolic dysfunction. We also discuss how gut microbiota influences the immunometabolism of different myeloid and lymphoid cell subsets and intestinal epithelial cells. Finally, we review the role of microbially produced metabolites, including short-chain fatty acids, lactate, succinate, bile acids, and amino acids, in reprogramming immune cell metabolism. We also discuss whether modulating gut microbiota function to regulate immunometabolic pathways could help restore immune homeostasis and reduce obesity-related complications.

## Introduction

Obesity has risen dramatically worldwide, driven largely by unhealthy, energy-dense dietary patterns that promote excess caloric intake and weight gain.^1^ The major metabolic complications of obesity include type 2 diabetes (T2D), steatotic liver disease, and cardiovascular disease. These disorders share a common mechanism in which chronic nutrient excess storage drives adipose tissue dysfunction, marked by elevated circulating fatty acids, leptin (an adipokine), and increased production of pro-inflammatory cytokines.^1^ Sustained immune activation leads to chronic inflammation and immune dysfunction, which, in turn, compromises tissue and systemic metabolic homeostasis.^2^ Beyond the diet, obesity‑associated shifts in the gut microbiota also contribute to immune activation, positioning the altered microbiota as a pivotal driver of the chronic inflammation characteristic of obesity.^3^

So far, most studies have focused on characterizing changes in the abundance and activation status of immune cell populations in obesity.^4^ However, a deeper understanding of immune dysfunction requires addressing the intracellular metabolic circuits that shape immune cells' behavior. Classic studies have shown, for instance, that feeding mice a fat-enriched diet alters the gut microbiota, thereby affecting the function of antigen-presenting cells in the differentiation of intestinal T helper (Th)17 cells, with a consequent impact on metabolic health.^5^ However, the metabolic reprogramming that these immune cells must undergo to achieve this phenotype has scarcely been investigated. This reflects the fact that, for several decades, immunology and metabolism evolved as largely separate disciplines; with few exceptions, immunologists viewed metabolic fluxes as merely processes to meet cellular energy demands, without considering their role in immune regulation.^6^ This paradigm has now shifted with the emergence of the field of immunometabolism, as numerous studies demonstrate that intracellular metabolism is a central regulator of innate and adaptive immunity, controlling immune cell fate and effector functions.^6^^,^^7^

In this review, we first summarize the principal metabolic pathways and master regulators that govern immunometabolism, providing an indispensable foundation for the concepts developed herein. Then, we present how obesity can reshape immune cell subsets, with a particular emphasis on the metabolic changes that drive their immune phenotype reprogramming. Finally, we highlight how obesogenic-driven changes in the gut microbiota impact immunometabolism and discuss the emerging role of microbially-derived metabolites in fine-tuning metabolic programs of immune cells.

## Intracellular metabolic pathways underpinning immunometabolism

ATP serves as the universal energy currency for all living cells, including those of the immune system, which require ATP for their development and activation. There are two main bioenergetic processes that generate ATP, which are closely connected: glycolysis, which occurs in the cytosol, and oxidative phosphorylation (OXPHOS), which takes place in the mitochondria. Glycolysis starts with the uptake of glucose by transporters, mainly glucose transporter (GLUT)1 in immune cells, and its subsequent processing in the cytosol to yield pyruvate, producing ATP and reducing NAD+ to NADH. Pyruvate is then internalized into the mitochondrial matrix, where it undergoes oxidative decarboxylation to form acetyl-CoA, which enters the tricarboxylic acid (TCA) cycle and feeds OXPHOS. However, mitochondrial energy production is not limited to glucose metabolism. A variety of nutrients, including fatty acids (FAs), amino acids, and ketone bodies, can be utilized for energy production. These substrates undergo processes such as fatty acid oxidation (FAO) and amino acid oxidation (AAO), as well as other catabolic pathways that ultimately feed the mitochondrial respiratory chain, producing ATP.^8^

The metabolic demands of immune cells are highly dynamic and tailored to their role in the immune response, distinguishing them from other cell types with more stable metabolic profiles. One event that increases the energy requirements of immune cells is activation, as occurs during bacterial infections, in response to dietary insults, or in conditions of gut barrier disruption.^9^ To meet this additional demand for ATP, activated immune cells readapt their metabolism to increase glucose uptake, boosting aerobic glycolysis (a phenomenon known as the Warburg effect), with consequent lactate production. Simultaneously, they undergo TCA cycle fragmentation, which ultimately limits mitochondrial OXPHOS.^10^ In other words, an activation of the immune response, as occurs in obesity, reprograms immune cells to ensure faster ATP production and a greater biosynthetic capacity for defense response and damage repair. However, compared with the mitochondrial pathway, aerobic glycolysis is not as efficient as OXPHOS for ATP production. Despite its low efficiency, glycolysis acts as a source of enzymatic cofactors (e.g., NADH) and metabolic intermediates that fuel the anabolic routes enhanced in activated immune cells. These include the pentose phosphate pathway (PPP) and fatty acid synthesis (FAS), which provide immune cells with key biomass components and the ability to generate effector molecules, such as cytokines and antibodies.^11^^,^^12^ Accordingly, *in vitro* studies show that activated cells exhibit increased glucose uptake and impaired performance in inhibition assays targeting glycolytic pathways.^13-15^ In addition, disruption of the TCA cycle leads to the accumulation of succinate and citrate, together with the induction of itaconate production, within immune cells. These metabolites contribute to the maintenance of the metabolic pathways governing pro-inflammatory responses.^16-18^ The excess of citrate provides essential carbon units for anabolic pathways such as FAS, which is a core pathway for the activation of inflammatory immune cells.^19^ An increase in cytoplasmic succinate stabilizes the hypoxia-inducible factor (HIF)-1α, a transcription factor that plays an essential role in cellular responses to hypoxia, including the transcription of genes related to inflammatory interleukins and those related to the glycolytic pathway (i.e., encoding for GLUT1), thus contributing to sustaining pro-inflammatory responses and glycolysis in activated cells.^20-22^ Succinate drives HIF-1α stabilization by direct inhibition of its major controllers (prolyl-hydroxylases) and indirectly by mitochondrial reactive species (mtROS) production through its oxidation.^20^ Itaconate, on the other hand, exerts a paradoxical function; unlike citrate and succinate, which accumulate early upon TCA cycle disruption, itaconate synthesis occurs mostly during a later phase of activation. It has a major role inhibiting succinate dehydrogenase (SDH), thus preventing succinate oxidation, which curtails mtROS production and stabilization of HIF-1α, being fundamental for inflammation resolution and avoidance of excessive cell damage along the inflammatory process.^18^^,^^23^

In contrast, immune cells responsible for functions such as tissue repair, homeostasis maintenance, and immunological memory show stronger dependence on mitochondrial metabolism. These cells rely primarily on the TCA cycle, OXPHOS, and/or FAO pathways to meet their energy requirements.^24-26^ The activation of these metabolic paths is accompanied by an increase in the active forms of enzymes, such as carbohydrate kinase–like protein (CARKL), phosphofructokinase-1/2 (PFK1/2), or pyruvate kinase isoform M2 (PKM2), which limit certain anabolic routes, like the PPP, while promoting mitochondrial metabolism.^27^^,^^28^ Certain bioactive and dietary compounds, such as resveratrol or curcumin, stimulate TCA and oxidative pathways.^29^^,^^30^ This stimulation leads to several beneficial effects, including the generation of immune tolerance, differentiation of memory cells, and promotion of type 2 immune responses.^29^^,^^30^ Conversely, when mitochondrial metabolic pathways or function are suppressed, there is a shift toward a more pronounced type 1 immune response, typically associated with inflammation and increased cell proliferation.^31^^,^^32^

## Master regulators of cellular energy homeostasis

**Figure 1.**
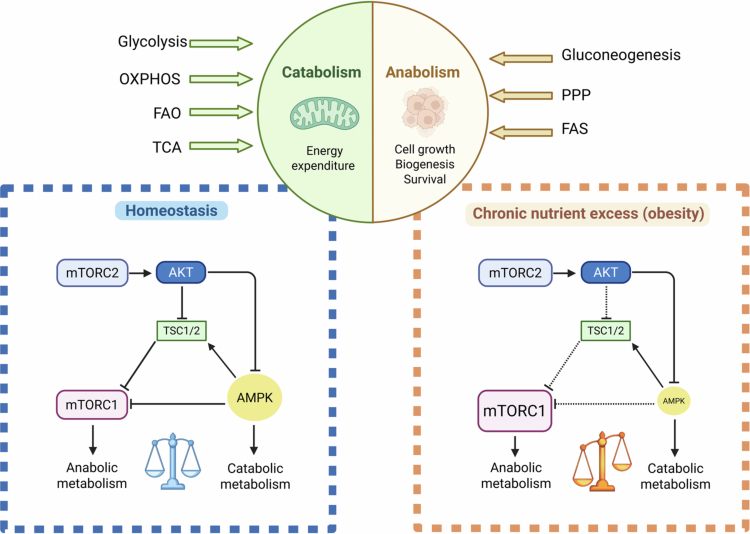
The AMPK/mTOR metabolic rheostat in immune cell homeostasis and obesity-induced dysregulation. Under physiological conditions (homeostasis), a dynamic equilibrium between AMPK and mTOR signaling maintains cellular fitness. AMPK senses low-energy states to promote catabolic pathways, such as fatty acid oxidation, ensuring immune quiescence. In contrast, mTOR facilitates anabolic and catabolic processes required for cell activation and proliferation. During chronic nutrient excess (obesity), this balance is disproportionately shifted toward constitutive mTOR activation. This metabolic reprogramming suppresses AMPK signaling, favoring a pro-inflammatory effector phenotype and contributing to the development of chronic low-grade systemic inflammation. Abbreviations: AMPK, AMP-activated protein kinase; mTOR, mammalian target of rapamycin.

The mammalian target of rapamycin (mTOR) and the AMP-activated protein kinase (AMPK) are key energy and nutrient sensors that regulate intracellular metabolism, exerting antagonistic roles and switching immune cells towards either anabolic or catabolic pathways, respectively^33^^,^^34^ (Figure 1).

mTOR is a serine/threonine protein kinase that is activated in response to nutrient and energy-rich environments (i.e., an obesogenic environment) and/or immune activation, promoting anabolic processes such as protein and lipid synthesis, cell growth, and survival. In particular, mTOR enhances proliferation and differentiation of effector T cells, pro-inflammatory macrophage activation, and dendritic cells (DCs) maturation and antigen presentation.^35^^,^^36^ mTOR is the core component of two distinct protein complexes: mTORC1 and mTORC2.^33^ When activated, mTORC1 regulates cellular functions to promote the transition from quiescence to anabolic metabolism and proliferation, while suppressing catabolic metabolism. mTORC2 affects cell survival and metabolism through the phosphorylation of protein kinase B (Akt), which, in turn, regulates glucose metabolism and cell survival pathways.^37^ Some effects of mTOR are mediated by the regulation of the NLR family pyrin domain-containing 3 (NLRP3) inflammasome. NLRP3 is an intracellular sensor that recognizes a broad range of microbial motifs, endogenous danger signals, and environmental irritants, leading to the formation and activation of the NLRP3 inflammasome, which is tightly linked to glycolysis.^38^

AMPK is a serine/threonine protein kinase composed of three polypeptide subunits (α, β, and γ).^39^ AMPK antagonizes mTOR in response to changes in the AMP/ATP ratio and nutrient deprivation.^33^ AMPK activation is associated with energy stress signaling and initiates catabolic reprogramming to meet ATP demands, ultimately reducing cell immunogenicity. In this context, AMPK can act as a direct inhibitor of mTOR, acetyl-CoA carboxylase 1 (ACC1), and the sterol regulatory element-binding protein 1c (SREBP1c). Consequently, AMPK activity reduces glycolysis and FAS, while promoting FAO and mitochondrial OXPHOS to maintain intracellular ATP levels.^33^ For example, in T cells, an enhanced AMPK activity stimulates pathways that facilitate their transition from an effector to a memory phenotype and promote Tregs, dampening excessive inflammation.^40^

## Metabolic rewiring of immune and epithelial cells in obesity

**Figure 2.**
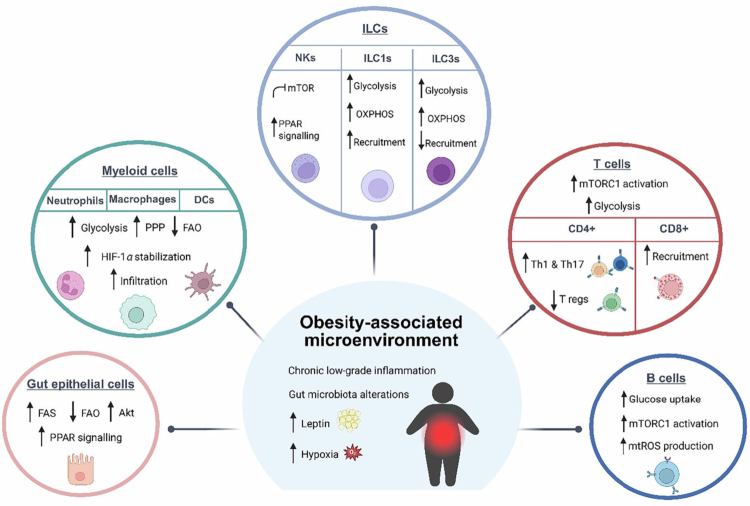
Systemic obesity-driven microenvironment and its impact on immune cell metabolic reprogramming. The obese microenvironment is characterized by chronic low-grade inflammation, gut microbiota dysbiosis, and elevated circulating levels of leptin and lipopolysaccharide (LPS). These systemic factors drive lineage-specific metabolic and functional shifts across various immune populations. In gut epithelial cells, there is a marked upregulation of protein kinase B (Akt) and peroxisome proliferator-activated receptor (PPAR) signaling alongside increased fatty acid synthesis (FAS). Myeloid cells undergo a metabolic switch toward glycolysis and the pentose phosphate pathway (PPP), supported by hypoxia-inducible factor (HIF)-1α stabilization and reduced fatty acid oxidation (FAO), which enhances tissue infiltration. Within the innate lymphoid cell (ILC) compartment, natural killer (NK) cells exhibit impaired mTOR signaling and altered PPAR pathways, while ILC1s and ILC3s show increased glycolysis and oxidative phosphorylation (OXPHOS), though with divergent recruitment patterns. The T cell pool is skewed toward a pro-inflammatory profile, with heightened mTORC1 activation and glycolytic flux favoring T helper (Th)1 and Th17 differentiation over Treg expansion, accompanied by an increase in CD8^+^ effector cells. Finally, B cells display enhanced glucose uptake, mTORC1 activation, and elevated mitochondrial ROS (mtROS) production, collectively contributing to the maintenance of meta-inflammation. Abbreviations: Akt, Protein kinase B; AMPK, AMP-activated protein kinase; FAO, Fatty acid oxidation; FAS, Fatty acid synthesis; HIF-1α, Hypoxia-inducible factor 1-alpha; ILC, Innate lymphoid cell; LPS, Lipopolysaccharide; mTORC1, Mammalian target of rapamycin complex 1; mtROS, Mitochondrial reactive oxygen species; NK, Natural killer; OXPHOS, Oxidative phosphorylation; PPAR, Peroxisome proliferator-activated receptor; PPP, Pentose phosphate pathway; Th, T helper cell; Treg, Regulatory T cell.

Obesity is associated with a persistent low-grade inflammatory state, known as meta-inflammation, characterized by sustained immune activation across tissues, such as adipose tissue, the liver, and the intestine. The development of new multi-omics approaches, such as single-cell RNA-sequencing, has expanded our understanding of obesity-associated immune imprinting across diverse immune cell subsets.^41^^,^^42^ For example, in the intestine of diet-induced obesity mice, it is observed an increased CD4^+^ T cell infiltration (mainly IFN-γ-producing Th1 and Th17 subsets), an augmentation of CD8^+^ T activation, and expression of chemokines involved in further recruitment of myeloid cells, as well as a decrease in cell-cell interaction in gut epithelial cells.^42^ In humans, similar immune alterations have been observed when comparing colon biopsies from obese and lean subjects. These studies have revealed increased pro-inflammatory recruitment of macrophages, Th1 cells, CD8^+^ T cells, and group 1 innate lymphoid cells (ILC1s),^43^^,^^44^ indicating a consistent shift towards immune activation under obesogenic conditions. These obesity-driven pro-inflammatory changes in the intestinal immune compartment mirror, to some extent, those reported in other metabolically active tissues. For instance, obese adipose tissue is similarly characterized by increased accumulation of pro-inflammatory macrophages, some of them with unique polarization states, such as lipid-associated macrophages,^41^ while within the T cell subsets, a decrease of Tregs and higher polarization towards T cell IFN-γ-producing phenotypes is observed.^41^^,^^43^ Ultimately, obesity creates a general lipotoxic and hypoxic environment that impairs immune surveillance and contributes to a sustained immune cell activation state, ultimately taking part in metabolic dysfunction. The impact of the pro-inflammatory tone driven by obesity in host metabolism has been extensively reviewed elsewhere.^44-46^ Here, by contrast, we focus on how the cellular metabolism of different immune subsets is rewired in an obesogenic environment and how this remodeling underpins the pro-inflammatory shift in these subsets (Figure 2).

### Gut epithelial cells

Epithelial cells are an essential component of gut barrier and play a crucial role in immune defense mainly through the secretion of mucus and antimicrobial peptides. They also undergo metabolic reprogramming in response to obesogenic stimuli, which can significantly alter their defensive functions and disrupt gut immunity. Hyperglycemia reprograms epithelial cells via a GLUT2-dependent transcriptional pathway, increasing intestinal barrier permeability.^47^ In particular, this process perturbs protein N-glycosylation by inducing a marked downregulation of genes critical for maintaining epithelial barrier function.^48^ This facilitates the influx of microbial products and foreign antigens into the systemic circulation, which can then reach peripheral organs, such as the liver and adipose tissue, thereby sustaining chronic low-grade inflammation and ultimately exacerbating insulin resistance, thereby linking local epithelial dysfunction to systemic metabolic impairment.

Intestinal stem cells (ISCs), which are undifferentiated cells found at the base of intestinal crypts, are also impacted by diet-induced obesity. In an obesity-promoting environment, ISCs exhibit cell dysfunction and hyperproliferation, as evidenced by accelerated differentiation and cell turnover.^49^^,^^50^ Specifically, Aliluev *et al.* observed that consumption of a diet enriched in fat and sugar elevates peroxisome proliferator-activated receptor (PPAR)γ signaling, SREBP1c-mediated lipogenesis, and Akt signaling, linking the increment of FAS and PPAR signaling to the hyperproliferation of the crypts.^50^ Other authors agreed that PPARγ contributes to the proliferation and dysfunction of ISCs exposed to a high-fat diet, linking this effect to the downstream FAO metabolic program.^49^ They also demonstrated that disruption of carnitine palmitoyl transferase 1 A (CPT1A), the FAO rate-limiting enzyme, facilitates the entry of FA into mitochondria, mitigating the phenotypic changes induced by the high-fat diet in ISCs.^49^

The metabolic changes occurring in differentiated epithelial cell subtypes have also been investigated. In Goblet cells, the NLRP6 inflammasome plays a crucial role in controlling mucus secretion.^51^ The small intestine of obese subjects shows decreased levels of α-defensin 5 and lysozyme, both antimicrobial peptides produced by Paneth cells.^52^ Palmitic acid, which is a component of obesogenic diets, also inhibits the production of α-defensins by Paneth cells.^53^ Specifically, there was a decreased expression of matrix metalloproteinase 7 (Mmp7), indicating that an obesogenic diet can alter the mTOR-Hes1-Atoh1-Mmp7-α-defensins axis.^48^

### Innate immune cells

#### Myeloid cells

Myeloid cells exhibit remarkable metabolic flexibility, undergoing profound metabolic shifts as they transition between various activation states to efficiently perform their functions. These subsets, which include macrophages, DCs, granulocytes, and mast cells, can rapidly adjust their metabolic profiles in response to environmental cues and functional demands. Obesogenic diets increase plasmatic oxidized low-density lipoprotein (oxLDL) levels, which can induce a reprogramming in myeloid progenitors via NLRP3 inflammasome activation.^54^ Notably, NLRP3^-/-^/Ldlr^-/-^ mice are protected from diet-induced immune reprogramming and systemic inflammation.^54^ Saturated FAs directly increase glycolytic rates and HIF-1α expression,^55^ which can additionally serve as enhancers of the NLRP3 inflammasome pathway.^56^ The specific deletion of the transcription factor HIF-1α in myeloid cells has been linked to reduced IL-1β production and increased expression of alternatively-activated macrophage markers in the adipose tissue of obese mice.^55^ This shift was attributed to a decrease in glycolytic rate in myeloid cells, directly driven by HIF-1α deficiency, and was hypothesized to result in alternative activation of the OXPHOS pathway.^55^ Moreover, Poblete *et al.* reported that IL-1 receptor-associated kinase M activity is responsible for the HIF-1α-induced adipose tissue malfunction, reinforcing the idea that myeloid cell HIF-1α contributes to adipose tissue growth and inflammation.^57^

Among myeloid cells, macrophage regulation is probably the most studied, and accumulating *in vitro* evidence indicates that metabolic rewiring drives differential inflammatory activation of macrophages, with alternatively-activated macrophages relying on OXPHOS and FAO, and pro-inflammatory macrophages relying on aerobic glycolysis and PPP pathways.^24^ However, opposite to this paradigm, Boutens *et al.* found that adipose tissue macrophages adopt a unique metabolic profile characterized by activation of multiple metabolic pathways, including both OXPHOS and glycolysis in obese mice.^58^ Along the same line, Wculek *et al.* challenged the previous assumption that pro-inflammatory macrophages rely primarily on glycolysis, showing that OXPHOS is critical for maintaining macrophages with high lipid-handling activity, including pro-inflammatory macrophages from the white adipose tissue.^59^ These authors propose that targeting OXPHOS could provide a selective therapeutic approach for treating obesity-related metabolic disorders.^59^ Overall, these studies highlight the enormous plasticity of these cells, which is likely fine-tuned by their metabolic balance.

Among granulocytic cells, neutrophils are the best studied in obesity. An increased infiltration of neutrophils into adipose tissue has recently been linked to obesity-associated microbiome signatures.^60^ Regarding intracellular metabolism, resting neutrophils are characterized by a high glycolytic rate and a relatively high level of FAO.^61^ However, upon a pro-inflammatory stimulation (i.e., lipopolysaccharide [LPS]), neutrophils dramatically increase glycolytic flux and gluconeogenesis while simultaneously reducing FAO.^61^ This metabolic adaptation ensures that neutrophils maintain sufficient energy reserves to fuel the higher energy demands of a pro-inflammatory response.^61^

#### Innate lymphoid cells (ILCs)

Obesity significantly alters ILCs' function and distribution in humans and animal models^62^; however, less is known about the effects of obesogenic diets on their cell metabolism. At steady state, the different ILC subsets rely mainly on OXPHOS for ATP production. However, when activated, ILCs undergo a metabolic shift, significantly increasing their rate of aerobic glycolysis.^63^ Besides, natural killers (NKs), which are members of the ILC1 subgroup, can display a more metabolically active phenotype, primarily manifested as increased glycolysis, OXPHOS, and mitochondrial membrane potential, elevated ATP synthesis, and increased glucose uptake.^64^ In NK cells, an obesogenic microenvironment induces PPAR expression and inhibits the mTOR pathway, ultimately driving intracellular lipid accumulation and impairing NK cytolytic function.^65^ Accordingly, the administration of FAs (palmitate and oleate) and PPAR agonists mimicked the effects of obesity and inhibited mTOR-mediated glycolysis, whereas PPAR inhibition restored NK cytotoxicity.^65^ Other authors found that another obesogenic factor, low-density lipoprotein (LDL), suppresses NK cell function through its interaction with dual specificity phosphatase 1 (DUSP1). DUSP1 acts as a potent anti-inflammatory agent by negatively regulating AMPK-induced nuclear factor kappa-light-chain-enhancer of activated B (NF-κB) cells.^66^ Therefore, obesity promotes the overactivation and recruitment of ILC1 in the adipose tissue and the gut, while limiting NK cytolytic function, contributing to the sustained inflammation.

Obesity also impairs ILC3s, which are a significant source of IL-22, a crucial cytokine for maintaining intestinal homeostasis and immune defense.^67^ At steady state, ILC3s rely on FA metabolism, while ILC3 proliferation and IL-22 production are dependent on the activation of the mTORC1-HIF-1α axis, enhancing glycolytic-OXPHOS dependent metabolism and retinoid-related orphan receptor (ROR)γt expression.^68^ Nonetheless, ILC3 activation requires mtROS production to stabilize RORγt and HIF-1α.^69^ Thus, the expectation would be that obesogenic diets, which create a low-oxygen environment (hypoxia), would increase ILC3 responses through a HIF-1α-dependent mechanism;^70^ however, a reduction in ILC3 responses has been reported in obesity, specifically their impaired ability to produce IL-22 in response to immune challenges or infection,^71^ showcasing our limited understanding of how obesity affects the intracellular metabolism of immune cell subsets, particularly ILC3.

### Adaptive immune cells

#### T cells

Diet-induced obesity perturbs T cell metabolism, leading to profound alterations in their bioenergetic profile and differentiation. The increased lipid availability driven by high-calorie diets consumption is hypothesized to be the primary factor driving changes in T cell metabolism and polarization.^8^ Indeed, the hyperleptinemia, characteristic of obesity, is a known key modulator of T cell metabolism.^72^ Studies in both humans and mouse models have revealed that leptin directly upregulates GLUT1 throughout mTORC1 activation.^73^ As a result, glycolysis increases in T cells, promoting CD4^+^ T cell differentiation towards the Th1 and Th17 phenotypes and the recruitment of CD8^+^ T cells, while reducing Treg cell numbers.^73^^,^^74^ Interestingly, Kiernan *et al.* showed that obesogenic-driven increases of oxidative metabolism and IFN-γ production in CD4^+^ T cells were mitigated when the leptin receptor was specifically depleted in T cells.^73^ In fact, leptin deficiency, caused either by malnourishment in humans or by genetic depletion in *ob/ob* mice, is associated with lower CD4^+^ and CD8^+^ T cell counts,^73^^,^^75^ which could be linked to lower glycolytic rates due to reduced mTORC1 stimulation, based on the current immunometabolic knowledge.

The increase in exposure to saturated FAs, specifically palmitate, in Western diets has been shown to cause metabolic stress, driving CD4^+^ T cells towards a pro-inflammatory effector memory phenotype via activation of the phosphoinositide 3-kinases (PI3K)-Akt pathway.^76^ Paradoxically, other authors attribute this inflammatory shift to augmented FAO metabolism, which is characteristically induced by AMPK-dependent activation and associated with an anti-inflammatory shift.^25^ In this sense, Akt has been shown to play a critical role in T cell activity and differentiation, acting as a master switch for substrate preference and downstream pathway activation.^77^ For instance, the transcription factor Foxp3 requires moderate or low levels of Akt activation.^78^ However, in obesity, Akt is highly and sustainedly activated,^79^ hindering Foxp3 induction and Treg differentiation,^80^ while its continuous activation dictates Th1 differentiation.^81^

#### B cells

In murine models of diet-induced obesity, B cell levels increase in visceral adipose tissue, which triggers T cell activation and the production of pro-inflammatory cytokines.^82^^,^^83^ This cascade further promotes pro-inflammatory responses in macrophages, contributing to insulin resistance and impaired glucose tolerance.^82^^,^^83^ Moreover, B cells undergo phenotypic changes, acquiring a pathogenic phenotype following exposure to an obesogenic diet. This was evidenced by the transference of splenic B cells from diet-induced obesity mice to obese B-cell-deficient mice, which significantly worsens glucose metabolism.^83^ Interestingly, this effect is not reproduced when B cells from mice fed a control diet are used for adoptive transfer experiments.^83^

Leptin, nutrient availability, and alterations in the gut microbiota have been suggested as mediators of altered B-cell function in obesity.^84^ Importantly, the AMPK pathway has been linked to preventing pro-inflammatory B cell activation, as it governs the transition of follicular B cells into mature quiescent cells.^85^ These mature B cells are characterized by enhanced glucose uptake, mitochondrial content (both number and size), and decreased mTORC1 activity compared to follicular B cells,^85^ characteristics that are counteracted by increased leptin levels. The study by Anderson et al. demonstrated that both rodents and humans consuming high-fat diets display increased mitochondrial H₂O₂ emission and a more oxidized cellular redox state without impairing mitochondrial respiration.^86^ Complementarily, Pal *et al.* revealed that diet-induced obesity alters mitochondrial metabolism in splenic B cells, leading to impaired respiratory capacity and elevated mtROS production, linking excess dietary fat to mitochondrial dysfunction in immune cells and immunometabolic dysregulation.^87^

## Gut microbiota remodeling in obesity and immune impacts

The intestine is the body's largest immune organ and is inhabited by trillions of bacteria that, in turn, influence immune function. It is also the first to be exposed to the diet, which also modulates the gut microbiota. Through these multidirectional interactions, the gut microbiota can moderate or exacerbate the intestinal immune system's response to diet, thereby influencing metabolic homeostasis at both intestinal and peripheral levels. In obesity, the gut microbiota is characterized by alterations in taxonomic composition, as documented in numerous studies^88^^,^^89^ and, in many cases, reductions in alpha diversity.^90^^,^^91^ At the genus and species level, obese individuals exhibit a decreased abundance of short-chain fatty acid (SCFA) producers (e. g., *Alistipes* spp., *Intestinimonas butyriciproducens,* and *Odoribacter splanchnicus*) and a depletion of bacteria that can support gut barrier integrity and immune homeostasis (e.g., *Akkermansia muciniphila* and *Bifidobacterium* spp.).^91^^,^^92^ Regarding microbial functionality, a recent meta‑analysis including 22,710 human microbiale metagenomes found a positive association between body mass index (BMI) and functional shifts in microbial metabolism. In particular, pathways involved in phosphatidylglycerol biosynthesis I and II, as well as glycogen biosynthesis I, which are in charge of storing excess carbon as an intracellular glycogen reserve, are increased in normal-weight individuals.^93^ These functional microbiome changes could affect the host's exposure to both gut bacterial ligands for immune cell receptors and metabolites that drive immune cell metabolic processes. Collectively, evidence supports a putative causal role for microbiome-derived taxonomic and functional pathways changes in obesity-associated meta-inflammation, as detailed in the next sections.

## Structural ligands of gut commensals shaping the immune landscape

The gut microbiome's role in immunity is primarily mediated by structural microbial cell components and microbially produced metabolites. Structural cell components act as microbe-associated molecular patterns (MAMPs), encompassing pathogen-associated molecular patterns (PAMPs), that are mainly recognized by innate immune cell receptors (e.g., Toll-like receptors [TLRs], nucleotide-binding oligomerization domain-containing proteins [NODs], etc.).^46^^,^^94^ TLRs' responses to microbes are among the most extensively studied; for instance, TLR4 senses LPS of Gram-negative bacteria, which has classically been associated with pro-inflammatory responses by enhancing glycolysis, PPP, and FAS pathways at the expense of OXPHOS and the Krebs cycle.^95^ However, in recent years, increasing evidence has highlighted the duality of LPS.^96^ Specifically, d'Hennezel *et al.* revealed that the collective LPS pool of the human gut microbiome acts as a TLR4 antagonist rather than an agonist.^97^ The study demonstrated that fecal LPS from healthy donors not only fails to trigger the TLR4-NF-κB signaling axis, but also competitively inhibits the pro-inflammatory cytokine response induced by *Escherichia coli* LPS.^97^ This suggests that the microbiome actively maintains intestinal homeostasis by silencing TLR4-mediated immunosurveillance, a mechanism essential for host tolerance toward its resident microbial load. A similar duality is observed in TLR2 responses. Beyond its role in pathogen recognition, TLR2 signaling in the intestinal epithelium has been shown to enhance tight junction protein function and promote mucosal integrity, further protecting the host-microbe symbiosis.^98^ Recently, we and others have described certain gut microbial members to protect against diet-induced obesity through TLR2-dependent mechanisms.^99^^,^^100^ We observed that *Phascolarctobacterium faecium* acts as a checkpoint inhibitor of the ILC1-induced inflammatory cascade in obesity through the enhancement of alternatively-activated macrophages polarization.^100^ However, the precise metabolic circuit orchestrating this macrophage rewiring has not yet been fully investigated. Recently, new links between TLR9 and obesity have been reported. TLR9 recognizes unmethylated DNA from bacteria and initiates type I IFN responses by immune cells.^101^ Wang *et al.* found that, in the absence of TLR9, specifically in B cells, mice were proner to the development of obesity.^102^ In addition, these authors showed that the absence of TLR9 in B cells led to reduced IL-10 in B cells, as well as to an altered gut microbiota composition accompanied by enhanced pro-inflammatory responses in the intestine.^102^ In this context, alterations in TLR9 signaling may impact the balance between AMPK and mTOR pathways, thereby modulating the shift between OXPHOS and glycolysis.

Beyond surface recognition, the internal detection of microbial structural components derived from gut commensals via NOD1 and NOD2 is increasingly recognized as a modulator of metabolic tone.^103^ These cytosolic receptors primarily sense fragments of peptidoglycan, triggering downstream signaling through the NF-κB and MAPK pathways.^104^ While these cascades are classically defined as drivers of pro-inflammatory gene expression, they are inherently energy-demanding processes that typically correlate with an upregulation of glycolytic flux to meet the immediate ATP requirements of the activated cell. However, it is worth noting that detailed immunometabolic profiling of NOD-specific activation remains sparse.

In summary, current literature has largely focused on the canonical activation of these signaling hubs, yet the precise metabolic shifts that occur following the sensing of structural microbial components remain an unresolved frontier in the context of immune cell function during obesity and deserve to be investigated in the future.

## Microbiota-derived metabolites: drivers of immune metabolic reprogramming

**Figure 3.**
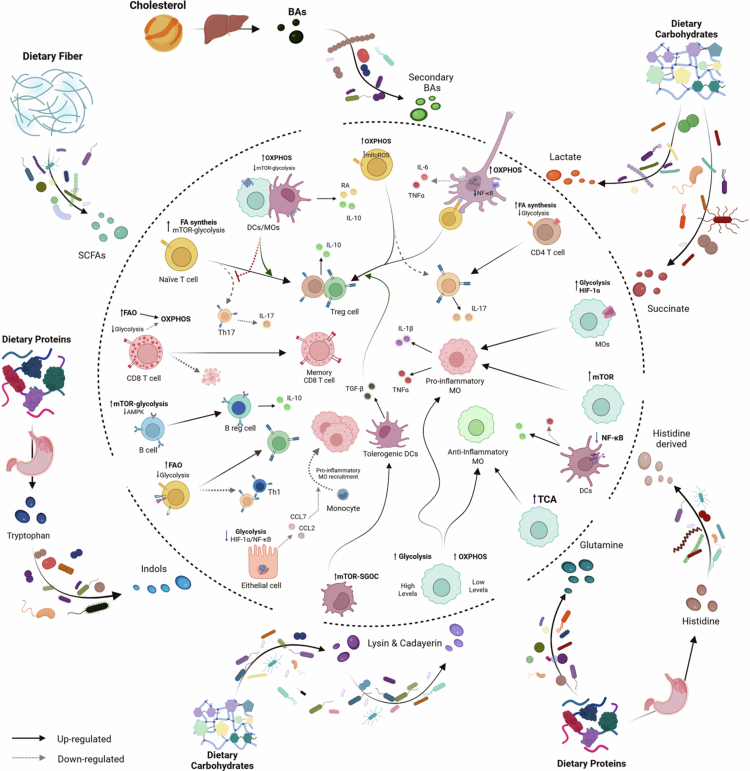
Microbiota-derived metabolites as key regulators of immunometabolism. Dietary components, including fiber, other carbohydrates, proteins, and cholesterol, are metabolized by the gut microbiota into bioactive metabolites that directly modulate the metabolic state and functional polarization of immune cells. Microbial-derived metabolites such as short-chain fatty acids (SCFAs), secondary bile acids (BAs), lactate, succinate, indole derivatives, amino acids (e.g., glutamine and histidine), and polyamines (e.g., lysine- and cadaverine-derived) act on epithelial cells and both innate and adaptive immune cells to reprogram core metabolic pathways, including glycolysis, fatty acid oxidation (FAO), oxidative phosphorylation (OXPHOS), the tricarboxylic acid (TCA) cycle, and fatty acid (FA) synthesis among others. In innate immune cells such as macrophages and dendritic cells, these metabolites are observed to shape activated states, inflammatory signaling (e.g., NF-κB, HIF-1α), and cytokine production, thereby promoting either pro-inflammatory or tolerogenic phenotypes. Similarly, in adaptive immune cells, metabolite-driven metabolic rewiring governs T and B cell differentiation and function, influencing fate decisions toward T helper (Th) (e.g., Th1, Th17) or regulatory T (Treg), memory or effector CD8⁺ T cell, and regulatory B cell responses. Overall, the figure highlights microbiota-derived metabolites as critical molecular intermediates of immunometabolic reprogramming, shaping immune cell fate and function. Abbreviations: AMPK, AMP-activated protein kinase; BA, bile acid; Breg, regulatory B cell; CCL, chemokine (C–C motif) ligand; DC, dendritic cell; FA, fatty acid; FAO, fatty acid oxidation; HIF-1α, hypoxia-inducible factor 1α; IL, interleukin; mtROS, mitochondrial reactive oxygen species; mTOR, mechanistic target of rapamycin; MO, macrophage; NF-κB, nuclear factor kappa B; OXPHOS, oxidative phosphorylation; RA, retinoic acid; SCFA, short-chain fatty acid; SGOC, serine, glycine, and one-carbon metabolism; TCA, tricarboxylic acid cycle; TGF-β, transforming growth factor beta; Th, T helper cell; TNF-α, tumor necrosis factor alpha; Treg, regulatory T cell.

Gut microbiome-produced metabolites can also activate the immune system with a downstream effect on immune cell metabolism and primarily exert their effects on intestinal tissues such as the colon and the ileum. Compared to structural microbial components, immunometabolic reprogramming driven by microbial metabolites has been more thoroughly characterized.^105^^,^^106^ However, most findings to date have been described in intestinal tissue, and their impact on immunometabolism in other metabolic tissues remains to be further explored. Below, we outline the effects of the main bacterial metabolites produced by gut commensals on immune cell metabolic reprogramming (Figure 3).

### Short-chain fatty acids (SCFAs)

SCFAs are produced by the microbiota and can be detected in the gut, liver, and bloodstream, as they can be absorbed across the intestinal epithelium. SCFAs have been implicated in the metabolic and epigenetic regulation of immune cells.^107^ Immune cells can sense SCFAs via G protein-coupled receptors (GPCRs) expressed on their surface.^108^ For instance, recognition of butyrate by GPCR109a initiates signaling that promotes anti-inflammatory responses in colonic macrophages and DCs and enables these cells to induce the differentiation of Treg and IL-10-producing T cells.^109^ Butyrate, but not acetate or propionate, reprograms macrophage metabolism toward OXPHOS and lipid metabolism, thereby promoting an anti-inflammatory phenotype,^110^ whose production has been attributed to key gut bacterial taxa such as *Faecalibacterium prausnitzii*, *Roseburia* and *Eubacterium* species, and whose abundance is often significantly reduced in human obesity cohorts.^111^ More recently, butyrate was shown to direct the differentiation of homeostatic colonic macrophages with strong antimicrobial activity, preventing bacterial dissemination beyond the intestinal barrier.^112^ At the cellular level, this protective response is mediated by inhibition of glycolysis and the mTOR pathway.^112^ Butyrate also increases FAO in activated CD8^+^ T cells, uncoupling OXPHOS from glycolytic inputs and enabling these cells to promote OXPHOS by utilizing glutamine and FA.^113^ These later findings, obtained from *in vitro* and *ex vivo* cell culture studies, are consistent with *in vivo* results, which demonstrate that butyrate administration to mice reduces systemic and tissue-specific inflammation, increases insulin sensitivity, and contributes to body weight regulation by exerting its primary effects on the colon, where it reaches its highest concentrations.^114^ However, this metabolite can be absorbed and transported to alternative organs, such as the liver, where it is largely metabolized. Despite this, its direct action in extraintestinal tissues remains poorly characterized. Nevertheless, reductions in inflammation in liver-related diseases have been reported, potentially as a consequence of reduced systemic inflammation produced by butyrate supplementation.^115^ Other SCFAs, such as pentanoate or acetate, stimulate the mTOR pathway in CD4^+^ T cells and B cells (via downregulation of AMPK activity) and glycolysis.^116^ These pathways governs the generation of T helper and regulatory T cells and utilizes SCFA-derived acetyl-CoA to fuel FA synthesis for antibody production, which may confer a protective effect in the context of obesity.

### Bile acids (BAs)

BAs are produced in the liver from cholesterol and conjugated with glycine or taurine. They are then secreted into the intestine, where gut microbes (e.g., *Bacteroides uniformis, Parabacteroides distasonis, Phocaeicola vulgatus*) modify them to generate the so-called secondary BAs, before they are partially reabsorbed and return to the liver.^117^ BAs and their derivatives can interact with membrane and nuclear receptors, such as the G protein-coupled bile acid receptor (TGR5), the farnesoid X receptor (FXR), and RORγt, thereby influencing cellular metabolism.^118^^,^^119^ However, in obesity and related diseases such as T2D and metabolic liver disease, various bacterial taxa have been implicated in the production of secondary bile acids. In T2D, taxa such as *Blautia* and *Ruminococcus* are reported to increase, whereas *Akkermansia* and *Faecalibacterium* were decreased; in metabolic liver disease, *Escherichia* and *Dorea* increased, whereas *Coprococcus* and *Prevotella* decreased. Thus, there is no consistency in taxonomic changes potentially responsible for bile acid production in the context of obesity and related diseases, likely due to inherent biological variability and functional redundancy.^120^

The impact of BA metabolites on immune cell subpopulations has been thoroughly reviewed^121^^,^^122^ and will not be discussed here. Instead, we provide emerging evidence on the role of secondary BAs in immune cell metabolism. A preclinical study described that two lithocholic acid (LCA) derivatives (3-oxoLCA and isoalloLCA) exhibit distinct regulatory effects on T cell subpopulations.^123^ Specifically, 3-oxoLCA inhibits the differentiation of Th17 cells by directly binding to the key transcription factor RORγt; whereas isoalloLCA increases the differentiation of Treg cells through the production of mtROS by mediating OXPHOS.^123^ Similarly, the secondary bile acid, 3-βhydroxydeoxycholic acid (isoDCA), is known to limit FXR-associated activity in DCs and induce an anti-inflammatory phenotype, reducing the production of inflammatory cytokines, such as TNF and IL-6.^119^^,^^124^ IsoDCA activates negative regulators of NF-κB and MAPK signaling, thereby promoting an anti-inflammatory phenotype in DCs and ultimately inducing Foxp3 and Treg cells.^124^ Importantly, the *in vivo* administration in mice and *in vitro* exposure to a *Bacteroides* strain engineered to produce isoDCA are enough to promote the generation of Tregs.^124^

### Lactate

Lactate is an active signaling molecule at both intracellular and extracellular levels, playing a critical role in immunometabolic processes, as it acts as a central intermediate of carbon metabolism. L-lactate is generated by host cells both at homeostasis and in pathological conditions (i.e., cancer and infections) and can also be produced by lactic acid bacteria and other commensal gut microbes during carbohydrate fermentation, whereas D-lactate is the predominant isomer synthesized by many gut microbiota species. Classically, it has been considered a dead-end waste product, but last year's studies have provided evidence of its major role in suppressing pro-inflammatory activated cells while promoting Tregs and alternatively-activated macrophages, as a secondary effect of aerobic glycolysis dysregulation.^125^ For instance, tolerogenic DCs create a lactate-rich environment that dysregulates aerobic glycolysis in T cells, increases FA synthesis and IL-17 production,^125^ while inducing Treg cell expansion.^126^

In the gut, D-lactate produced by commensal bacteria is absorbed into the portal circulation, where it can shape immune cell trafficking, including neutrophil homecoming, thereby enhancing host defense.^127^ While this may be beneficial in an infection context,^128^ in obesity, lactate can be detrimental, further contributing to systemic inflammation.^127^ For example, Fang *et al.* demonstrated that microbiota-derived D-lactate accumulates in the circulation of obese mice and exhibits higher immunogenicity than the L-isomer, thereby promoting Kupffer cell activation and hepatic neutrophil recruitment, further contributing to systemic inflammation.^127^ Nonetheless, lactate can also exert context-dependent protective effects, as it has been associated with greater beige fat activation in mouse models of obesity.^129^ Another example is its role as fuel for the TCA cycle to drive histone H3K27 acetylation, thereby inducing immunosuppressive transcriptional programs that induce long-term immunosuppression and endotoxin tolerance, including LPS.^130^

### Succinate

Succinate is a metabolite resulting from microbial carbohydrate fermentation, which plays a role in epithelial and immune cell metabolism. In the context of obesity, elevated plasma succinate levels have been detected, along with an increased ratio of succinate-producing bacteria (i.e., *Prevotellaceae, Veillonellaceae*) to succinate-consuming (i.e., *Phascolarctobacterium, Odoribacteraceae, Clostridaceae*), supporting its microbial origin.^131^ Epithelial cells and macrophages internalize extracellular succinate via SLC13 transporters.^132^ In an inflammatory context, the uptake of extracellular succinate by macrophages can increase nitric oxide (NO) production, indicating sustained activation of the inducible nitric oxide synthase (iNOS) pathway, which is accompanied by increased glycolysis and mitochondrial activity.^21^ Additionally, in macrophages, cytosolic accumulation of succinate stabilizes HIF-1α, as stated before, which contributes to a glycolytic metabolic shift and, thus, to a pro-inflammatory activation through the succinate-succinate receptor (SUCNR)1 signaling axis, ultimately promoting IL-1β production.^21^ Therefore, succinate derived from obesity-linked microbiota profiles emerges as an important immunometabolic cue that can reprogram macrophage function, amplifying and sustaining inflammation.

### Amino acid-related metabolites

#### Tryptophan-derived metabolites

Tryptophan plays a significant role in immune cell metabolism. It can be metabolized through three primary pathways: the kynurenine pathway, the serotonin pathway, and the indole pathway. The first two pathways occur within mammalian cells, while the indole pathway is facilitated by gut microbiota.^133^ Specifically, commensal microorganisms catabolize tryptophan into a diverse array of bioactive indoles. For instance, indole-3-acetic acid (IAA) is synthesized by species such as *Lactobacillus reuteri,*^134^ while indole-3-lactic acid (ILA) is typically produced by members of the genus *Bifidobacterium* and other lactic acid bacteria, including *Lactobacillus plantarum.*^135^^,^^136^ Furthermore, specialized anaerobes like *Clostridium sporogenes* are responsible for the production of indole-3-propionic acid (IPA).^137^ In particular, reduced IPA levels are a hallmark of obesity and related metabolic disorders, being inversely correlated with BMI, triglycerides, and inflammation, and positively correlated with high-density lipoprotein (HDL) levels.^138^ In a recent study, Qing *et al.* demonstrated that IPA promotes mitochondrial respiration in CD4^+^ T cells by increasing FAO and AAO production, thus inhibiting the glycolytic rate, and therefore interfering with Th1 and Th17 differentiation without affecting Treg proportions.^139^ ILA has also been reported to exert a protective role on intestinal epithelial barrier by activating the aryl-hydrocarbon receptor (AhR)-nuclear factor erythroid 2-related factor 2 (Nrf2) pathway and downregulating claudin-2 expression.^140^ Moreover, ILA has been shown to suppress glycolysis and the NF-κB and HIF-1α signaling pathways via AhR, leading to decreased CCL2/7 production by epithelial cells and, consequently, reduced recruitment of pro-inflammatory macrophages.^141^

#### Glutamine

Glutamine is an ubiquitous amino acid that can be synthesized and used by many environmental and gut bacteria (e.g.*, Escherichia* spp*., Brevibacterium* spp.).^142^ Regarding gut microbiota-derived glutamine, a beneficial effect has been observed in cases of liver ischemia.^143^ Elevated glutamine levels in the gut increase α-ketoglutarate levels in the blood, which promote anti-inflammatory responses in macrophages by fueling the TCA cycle; the increased number of alternatively-activated macrophages can repair hepatic injury *in vivo.*^143^ Despite this promising mechanistic study, there is a notable scarcity of literature exploring the specific contribution of microbial glutamine to systemic immunometabolism. One recent study underscored the relevance of this metabolic axis, showing that glutamate, derived from glutamine through glutaminase-mediated deamidation, can also reprogram bone marrow-derived macrophages.^144^ Specifically, glutamate stabilizes HIF-1α, which transcriptionally activates WNT3 to promote YAP1-dependent hepatocyte proliferation, thereby boosting liver regeneration.^144^ In the gut, glutamate can be synthesized by various commensals from glutamine, most notably *Bacteroides thetaiotaomicron*, which utilizes it as a central hub for nitrogen metabolism.^145^ Interestingly, while this species possess the enzymatic machinery for glutamate production, some studies report that its abundance is markedly depleted in individuals with obesity, a condition characterized by systemic glutamate dysregulation.^145^ This might suggest that *B. thetaiotaomicron* serves as a key orchestrator of glutamate flux; its reduction may impair the fine-tuned amino acid signaling required for the macrophage-mediated tissue repair mechanisms described above.

#### Lysine and cadaverine

L-lysine is an essential amino acid that humans cannot produce and must obtain from the diet. It plays key physiological roles in growth, bone health, calcium absorption, and the maintenance of lean body mass.^146^ Its metabolism influences energy and nutrient signaling via the Akt/mTOR pathway and generates acetyl-CoA through saccharopine and 2-aminoadipic acid,^147^ potentially affecting lipid synthesis and metabolic homeostasis in obesity. Additionally, commensal gut bacteria, such as *Enterobacter ludwigii*, can synthesize lysine via the diaminopimelate (DAP) pathway, thereby providing an additional source. Tang *et al.* recently described that lysine produced microbially in the gut stimulated the serine, glycine, and one-carbon metabolism pathways in DCs via the AMPK/acetyl-CoA/mTOR signaling axis.^148^ This shift leads to epigenetic changes that enrich the *Tgfb* and *Stat3* gene promoter activities and support an immune-tolerant DC phenotype.^148^ In the context of obesity, where chronic low-grade inflammation is prevalent, one can hypothesize that lysine-driven modulation of DCs' metabolism and function could help restrain excessive inflammatory responses and support immune homeostasis.

Recently, cadaverine, a biogenic amine formed by the decarboxylation of lysine by gut microbes, has been shown to exert context- and concentration-dependent immunometabolic effects on macrophages.^149^ De Oliveira *et al.* reported that under basal conditions, it is internalized by lysine transporters in macrophages, thereby activating the thioredoxin system, promoting mitochondrial respiration, and supporting an anti-inflammatory, regulatory phenotype. In the inflammatory context of irritable bowel disease, cadaverine stimulates the aconitate decarboxylase 1 (Acod1) pathway, inducing itaconate production and activating Nrf2, also promoting an immunoregulatory phenotype in macrophages and maintaining mitochondrial function. Nevertheless, at high concentrations, cadaverine signals via the histamine 4 receptor, triggering a glycolytic metabolism, pro-inflammatory macrophage activity, and enhanced inflammation.^149^ These findings highlight cadaverine as a key microbiota-derived metabolite capable of shaping macrophage immunometabolism and modulating inflammatory responses in a context-dependent manner across health and disease.^149^

#### Histidine

Traditionally, histamine has been the most extensively studied histidine-derived bacterial metabolite due to its well-known effects on host immunity, vascular tone, and epithelial barrier function.^150^ In DCs, histamine derived from the microbiota interacts with the histamine 2 receptor (H2R), dampening LPS-induced activation and reducing NF-κB/AP-1 activity and MAPK phosphorylation as well as the induction of pro-inflammatory cytokines and chemokines, while enhancing IL-10 production.^151^ Consistent with this, *Lactobacillus rhamnosus*, which produces histamine, suppresses the production of multiple cytokines in Peyer's patches in wild-type mice but not in H2R-deficient mice, indicating that its immunomodulatory effects depend on H2R signaling.^151^ Imidazole propionate (ImP), another gut microbiota-derived metabolite, has been reported to be elevated in the portal blood of individuals with T2D and to impair insulin signaling by activating the p38γ/p62/mTORC1 pathway.^151^ Although this work focused on hepatic rather than immune cell metabolism, recent studies have implicated ImP in the development of atherosclerosis through immune mechanisms.^152^ In particular, Mastrangelo *et al.* found that ImP triggers S6 phosphorylation (an indicator of mTOR activation) and induces pro-inflammatory cytokine production in macrophages, thereby promoting atherosclerosis.^152^ Interestingly, blocking the ImP-I1R axis inhibited the development of atherosclerosis induced by ImP or a high-cholesterol diet in mice, opening new avenues for atherosclerosis therapy.^152^ Similarly, in the context of obesity, gut microbes capable of producing histidine-derived metabolites could contribute to a sustained inflammatory status by promoting glycolytic metabolism in immune cells.

## Conclusions and future perspectives

Immune cell metabolism is a highly adaptive and complex process, shaped by specific environmental cues and bioenergetic requirements that determine immune cell fate and function. Obesity is associated with profound metabolic reprogramming of immune cells, which remains poorly understood. The gut microbiota provides both structural components and a diverse array of bioactive metabolites from carbohydrate and amino acid metabolism, which can impact the metabolic programming of immune cells. Nonetheless, future research is needed to dissect how these microbial signals directly reshape immune cell bioenergetics and function, and to establish causality in the context of obesity. While some evidence exists regarding the immunometabolic effects of microbiota-derived metabolites, their production patterns in obesity remain unclear, and more importantly, these changes cannot yet be reliably linked to specific taxonomic compositional and functional alterations. Deciphering these complex interactions through novel multi-omics approaches and loss-of-function studies is essential to transition from descriptive observations to a mechanistic blueprint of cellular rewiring, a prerequisite for effectively restoring metabolic and immune homeostasis.

## Data Availability

Original data has not been generated in this study.

## Abbreviations

AAO: amino acid oxidation
ACC1: acetyl-CoA carboxylase 1
Acod1: aconitate decarboxylase 1
Ahr: aryl-hydrocarbon receptor
Akt: protein kinase B
AMPK: 5ʹ adenosine monophosphate-activated protein kinase
ATP: adenosine triphosphate
BMI: body mass index
CARKL: carbohydrate kinase–like protein
CPT1A: carnitine palmitoyl transferase 1 A
DCs: dendritic cells
FAs: fatty acids
FAO: fatty acid oxidation
FAS: fatty acid synthesis
FXR: farnesoid X receptor
GLUT: glucose transporter
GPCRs: G protein-coupled receptors
H2R: histamine 2 receptor
HDL: high-density lipoprotein
HIF-1 α: Hypoxia-inducible factor -1α
IAA: indole-3-acetic acid
Ig: immunoglobulin
ILA: indole-3-lactic acid
ILCs: innate lymphoid cells
ImP: imidazole propionate
iNOS: inducible nitric oxide synthase
IPA: indole-3-propionic acid
ISCs: intestinal stem cells
IsoDCA: 3-βhydroxydeoxycholic acid
LCA: lithocholic acid
LDL: low-density lipoprotein
LPS: lipopolysaccharide
MAMPs: microbe-associated molecular patterns
MAPK: mitogen-activated protein kinase
mTOR: mammalian target of rapamycin
mtROS: mitochondrial reactive oxygen species
NAD: nicotinamide adenine dinucleotide
NADH: reduced form of nicotinamide adenine dinucleotide
NF-κB: nuclear factor kappa-light-chain-enhancer of activated B cells
NK: natural killers
NLRP3: NLR family pyrin domain-containing 3
NO: nitric oxide
NOD: nucleotide-binding oligomerization domain
Nrf2: nuclear factor erythroid 2-related factor 2
oxLDL: oxidized low-density lipoproteins
OXPHOS: oxidative phosphorylation
PAMPs: pathogen-associated molecular patterns
PFK1/2: phosphofructokinase-1/2
PI3K-Akt: phosphoinositide 3-kinase/protein kinase B
PKM2: pyruvate kinase isoform M2
PPAR: peroxisome proliferator-activated receptor
PPP: pentose phosphate pathway
PRRs: pattern recognition receptors
RORγt: retinoic acid receptor-related orphan receptor gamma t
SCFAs: short-chain fatty acids
SDH: succinate dehydrogenase
SREBP1c: sterol regulatory element-binding protein 1c
T2D: type 2 diabetes
TCA: tricarboxylic acid
TGR5: G protein-coupled bile acid receptor
Th: T helper
TLRs: toll-like receptors
Tregs: regulatory T cells
